# Post-neoadjuvant treatment pancreatic cancer resectability and outcome prediction using CT, ^18^F-FDG PET/MRI and CA 19–9

**DOI:** 10.1186/s40644-023-00565-8

**Published:** 2023-05-22

**Authors:** Jeongin Yoo, Jeong Min Lee, Ijin Joo, Dong Ho Lee, Jeong Hee Yoon, Mi Hye Yu, Jin-Young Jang, Sang Hyub Lee

**Affiliations:** 1 Department of Radiology, Seoul National University Hospital, Seoul, Korea; 2 Department of Radiology, Seoul National University College of Medicine, Seoul, Korea; 3 Institute of Radiation Medicine, Seoul National University College of Medicine, Seoul, Korea; 4 Department of Radiology, Konkuk University School of Medicine, Seoul, Korea; 5 Department of General Surgery, Seoul National University College of Medicine, Seoul, Korea; 6 Department of Internal Medicine, Seoul National University College of Medicine, Seoul, Korea

**Keywords:** Pancreatic cancer, Neoadjuvant therapy, Computed tomography, Postiron emission tomography/magnetic resonance imaging, Carbohydrate antigen 19–9, Resectability

## Abstract

**Background:**

CT prediction of resectability and prognosis following neoadjuvant treatment (NAT) in patients with pancreatic ductal adenocarcinoma (PDAC) remains challenging. This study aims to determine whether addition of ^18^F-fluorodeoxyglucose (FDG) postiron emission tomography (PET)/MRI and carbohydrate antigen (CA) 19–9 to contrast-enhanced CT (CECT) can improve accuracy of predicting resectability compared to CECT alone and predict prognosis in PDAC patients after NAT.

**Methods:**

In this retrospective study, 120 PDAC patients (65 women; mean age, 66.7 years [standard deviation, 8.4]) underwent CECT, PET/MRI, and CA 19–9 examinations after NAT between January 2013 and June 2021. Three board-certified radiologists independently rated the overall resectability on a 5-point scale (score 5, definitely resectable) in three sessions (session 1, CECT; 2, CECT plus PET/MRI─no FDG avidity and no diffusion restriction at tumor-vessel contact indicated modification of CECT scores to ≥ 3; 3, CECT plus PET plus CA 19–9─no FDG avidity at tumor-vessel contact and normalized CA 19–9 indicated modification of CECT scores to ≥ 3). Jackknife free-response receiver operating characteristic method and generalized estimating equations were used to compare pooled area under the curve (AUC), sensitivity, and specificity of three sessions. Predictors for recurrence-free survival (RFS) were assessed using Cox regression analyses.

**Results:**

Each session showed different pooled AUC (session 1 vs. 2 vs. 3, 0.853 vs. 0.873 vs. 0.874, *p* = 0.026), sensitivity (66.2% [137/207] vs. 86.0% [178/207] vs. 84.5% [175/207], *p* < 0.001) and specificity (67.3% [103/153] vs. 58.8% [90/153] vs. 60.1% [92/153], *p* = 0.048). According to pairwise comparison, specificity of CECT plus PET/MRI was lower than that of CECT alone (adjusted *p* = 0.042), while there was no significant difference in specificity between CECT alone and CECT plus PET plus CA 19–9 (adjusted *p* = 0.081). Twenty-eight of 69 patients (40.6%) with R0 resection experienced tumor recurrence (mean follow-up, 18.0 months). FDG avidity at tumor-vessel contact on post-NAT PET (HR = 4.37, *p* = 0.033) and pathologically confirmed vascular invasion (HR = 5.36, *p* = 0.004) predicted RFS.

**Conclusion:**

Combination of CECT, PET and CA 19–9 increased area under the curve and sensitivity for determining resectability, compared to CECT alone, without compromising the specificity. Furthermore, ^18^F-FDG avidity at tumor-vessel contact on post-NAT PET predicted RFS.

**Supplementary Information:**

The online version contains supplementary material available at 10.1186/s40644-023-00565-8.

## Introduction

Neoadjuvant treatment (NAT) using chemotherapy with or without additional radiation is the current accepted standard of care for patients with borderline resectable and locally advanced pancreatic ductal adenocarcinoma (PDAC) [1], since it holds promise for downstaging PDAC and enhancing the rate of R0 resection [2–4]. The National Comprehensive Cancer Network (NCCN) guideline currently recommends the use of multi-detector CT as the preferred imaging modality to be performed at presentation and 4 weeks before surgery for staging [5]. However, it is challenging to assess the therapeutic response of PDAC to NAT and determine candidates for surgical resection using contrast-enhanced CT (CECT) [4, 6]. This could be due to the fact that NAT-induced tumor cell injury is mainly reflected by "isovolumetric" tissue replacement through fibrosis rather than volume loss [7]. Additionally, NAT induces necrosis, edema, and inflammation of the tumor, which interferes with radiologic evaluation of tumor regression [8, 9]. Therefore, several studies have demonstrated that CECT showed suboptimal diagnostic performances in the context of staging after NAT [10, 11]. Consequently, the need for an ideal staging tool to assess resectability and a prognostic imaging biomarker after NAT remains unmet [12].

The implementation of a whole-body integrated PET/MR imaging system has shown promising results in the diagnosis, staging, and monitoring of various types of oncologic disease, notably pancreatic cancer [13, 14]. Given the advantages that MRI and PET imaging can offer separately, integrated PET/MRI can potentially be superior to each modality alone. The technique would have the advantage of high soft tissue contrast of MRI and metabolic information from PET [15–17]. A previous preliminary study demonstrated the usefulness of PET/MRI in pancreatic cancer, showing diagnostic performance similar to that of PET/CT plus CECT in preoperative evaluation of resectability [16]. Other studies reported that diffusion or metabolic parameters could predict resectability and prognosis after NAT [18, 19]. Recent NCCN guidelines recommend the use of both imaging studies and carbohydrate antigen (CA) 19–9 levels for consideration of resection following NAT, since several studies have demonstrated the prognostic role of CA 19–9 for resectability and survival of patients with PDAC after NAT [20, 21]. Therefore, we hypothesized that multiparametric information provided by PET/MRI and change in CA 19–9 level would correlate with resectability and/or post-resection survival in patients with PDAC after NAT.

This study aimed to determine whether addition of ^18^F-fluorodeoxyglucose (FDG) PET/MRI and CA 19–9 to CECT could improve the accuracy of predicting resectability compared to CECT alone and predict prognosis in patients who underwent NAT for PDAC.

## Methods

This retrospective study was approved by our institutional review board, and the requirement for signed informed consent was waived due to the retrospective study design.

### Patients

**Fig. 1:**
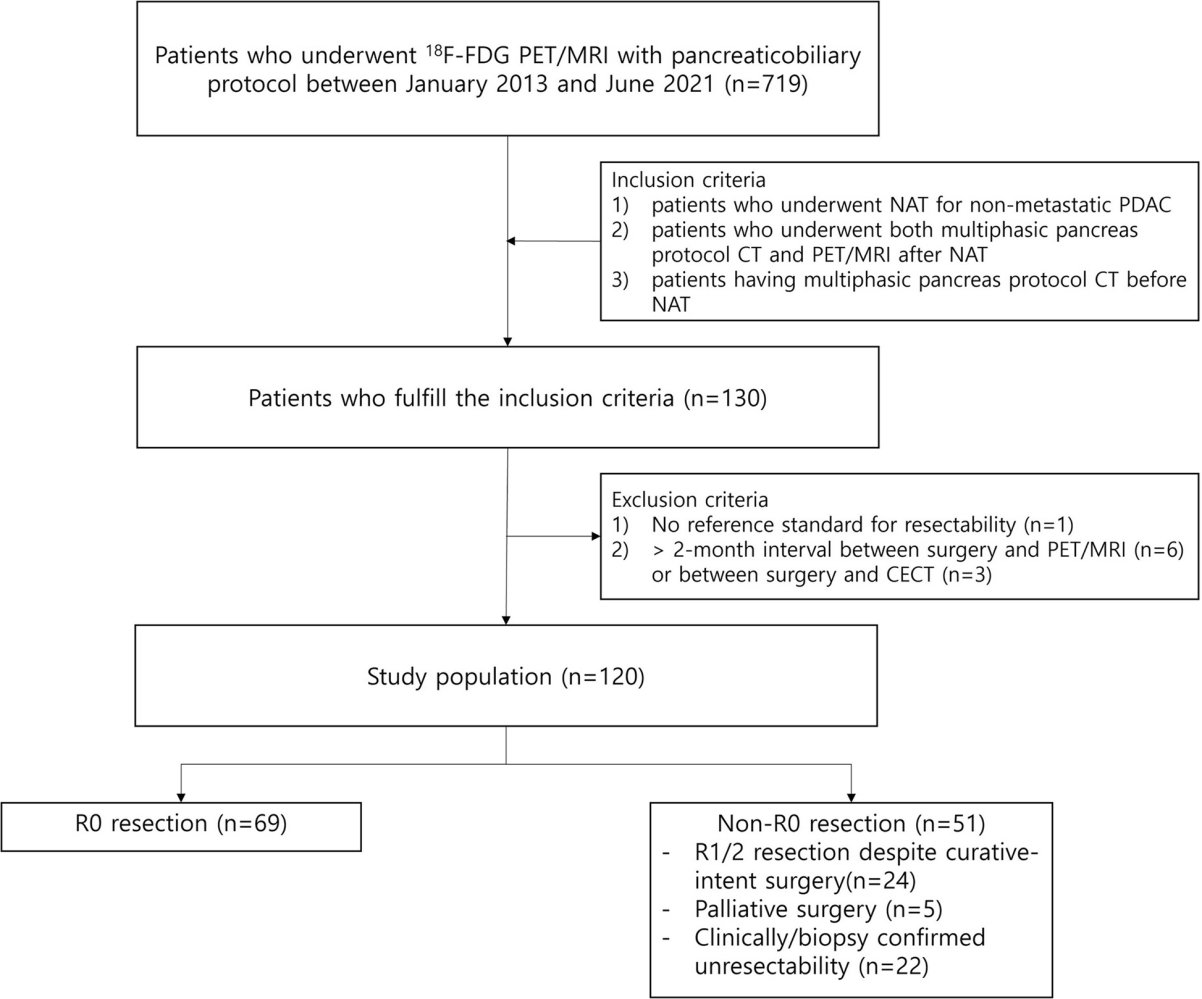
Flow diagram showing patient enrollment process. FDG, fluorodeoxyglucose; PET, positron emission tomography; NAT, neoadjuvant therapy; PDAC, pancreatic ductal adenocarcinoma; CA 19–9, carbohydrate antigen 19–9

The radiologic database at our institution showed that 719 patients underwent whole-body PET/MRI including a dedicated pancreatic protocol MRI between January 2013 and June 2021 (Fig. 1). The inclusion criteria were: 1) patients who underwent NAT for pathologically confirmed PDAC without distant metastasis, which was determined by CT, 2) those who underwent both multiphasic pancreas protocol CT and PET/MRI for response evaluation and/or decision of resectability after NAT, and 3) those who had multiphasic pancreas protocol CT before NAT. Among 130 patients who met the inclusion criteria, 10 patients were excluded due to: 1) no reference standard for resectability (*n* = 1) and 2) > 2-month interval between surgery and PET/MRI (*n* = 6) or between surgery and CECT (*n* = 3). Thus, a total of 120 patients were included. All 120 patients had both initial and post-NAT CA 19–9 levels.

### Neoadjuvant chemoradiation therapy

NAT regimens included FOLFIRINOX (a combination of 5-fluorouracil, oxaliplatin, irinotecan, and leucovorin) or gemcitabine with or without radiotherapy (FOLFIRINOX with radiotherapy [*n* = 76], FOLFIRINOX only [*n* = 36], and gemcitabine with radiotherapy [*n* = 8]) (Supplementary material 1).

### Image acquisition

All patients underwent pancreas protocol CT scans, including precontrast, arterial, and portal venous phases, using MDCT scanners with 16–320 channels (Supplementary material 2). CT acquisition parameters were as follows: tube voltage, 120 kVp; tube current, 150–250 mAs; slice thickness, 2–3 mm; reconstruction interval, 0.6–5 mm; pitch, 0.9–1; rotation time, 0.5–1 s. In general, CECT scans were obtained after an injection of iobitridol (Xenetics 350, Guerbet) based on body weight (525 mg I/kg, 1.5 mL/kg) for 35 s (injection rate, 2.0–5.0 mL) with an automatic power injector (Stellant Dual, Medrad) followed by a 30-mL saline flush.

All PET/MRI examinations were performed using a 3 T simultaneous PET/MRI scanner (Biograph mMR, Siemens Healthineers), and consisted of whole-body PET/MRI and dedicated pancreatic protocol MRI using extracellular contrast media (Supplementary material 3). The MR imaging protocol included the following sequences: Breath-hold transverse T2-weighted imaging with a single-shot turbo spin echo with fat saturation, T1-weighted in- and opposed-phase spoiled three-dimensional (3D) gradient-echo (GRE) sequence, breath-hold two-dimensional MR cholangiopancreatography (MRCP), respiratory triggered 3D MRCP, diffusion-weighted imaging (DWI) using a single-shot echo-planar imaging pulse sequence with b values of 0, 50, 400 and 800 s/mm^2^ using respiratory triggering, and a breath-hold T1-weighted fat-suppressed GRE sequence. An apparent diffusion coefficient map was calculated from DWI. We obained dynamic contrast-enahnced MR images that covered the area from the liver dome to the third portion of the duodenum, with the administration of a 7.5 mL 1.0 M gadobutrol (Gadovist; Bayer Healthcare) at a dose of 0.1 mmol/kg of body weight at an injection rate of 2 mL/s. The arterial phase imaging was obtained 8 s after arrival of contrast material in the distal thoracic aorta. Portal venous and delayed phase images were acquired approximately 60 s, 2, 3, and 5 min after the contrast material administration.

### Image interpretation

Three board-certified radiologists (with 14, 15, and 15 years’ experience, respectively, in abdominal imaging, including PET/MRI) independently reviewed the CECT images first, followed by CECT plus PET/MRI and CECT plus PET plus CA 19–9 within > 1-month interval to minimize recall bias. The reviewers were blinded to the clinical-surgical-pathologic results, except that the patients underwent NAT for PDAC. In each session, baseline CECT images were provided together. In addition, the reviewers were allowed to utilize MRI of PET/MRI as a point of reference when evaluating tumor-vessel contact on DWI or PET of PET/MRI.

#### Post-NAT CT resectability criteria

Resectability criteria were based on conventional criteria at the time of diagnosis according to NCCN guideline [1]. Interval development of non-enhancing peritumoral infiltration was not considered in resectability assessment [1, 22], since NAT could induce edema or fibrosis [11]. Reviewers determined local resectability on a 5-point confidence scale [score 1 (definitely unresectable) to 5 (definitely resectable)], considering local tumor extent and tumor-vessel relationship (Supplementary material 4) [1, 22].

Likelihood of distant metastasis was scored on a 3-point scale [1 (metastasis), 2 (indeterminate), and 3 (no metastasis)]. On CECT, detection of liver metastases relied on identifying hypovascular solid masses exhibiting rim enhancement, which are indicative of characteristic features. Lymph node metastasis was determined on the basis of short-axis diameter ≥ 10 mm and morphological features such as internal necrosis or the degree of enhancement [22, 23]. On PET/MRI, diffusion restriction and FDG uptake were used to characterize the lesions [19]. Overall tumor resectability was scored between 1 (definitely unresectable) and 5 (definitely resectable), considering both local unresectability and distant metastasis. Reviewers additionally categorized all the cases into locally advanced or metastatic, borderline resectable, and resectable tumors according to the NCCN guidelines [1].

#### Post-NAT CECT plus PET/MRI resectability criteria

Diffusion restriction on diffusion-weighted imaging (DWI) or FDG avidity on PET imaging was qualitatively determined at tumor-vessel contact in comparison to the adjacent pancreatic parenchyma. In cases with CECT resectability score 1 to 4, when there was no FDG avidity and no diffusion restriction at tumor-vessel contact, the reviewers upward modified the score to 3 or higher. CECT resectability score was modified downward in cases with distant metastasis determined on PET/MRI.

#### Post-NAT CECT plus PET imaging plus CA 19–9 resectability criteria

In cases with CECT resectability score 1 to 4, when there was no FDG avidity at tumor-vessel contact and normalized CA 19–9 levels (> 37 U/mL at baseline, reduced to < 37 U/mL during follow-up) [23], the reviewers upward modified the score to 3 or higher. CECT resectability score was modified downward in cases with distant metastasis determined on PET imaging.

Consensus was reached when at least two reviewers assigned the same score. Discrepancies among the three reviewers were resolved by a fourth reviewer (with 26 years of experience). Additionally, one board-certified radiologist with 8 years of experience measured the maximum standardized uptake value and apparent diffusion coefficient value on PET/MRI at tumor-vessel contact in all patients.

### Standard of reference

Reference standard for tumor resectability was based on the clinical-surgical-pathologic findings. In patients who had undergone surgery, resectability was classified according to surgical records and pathology reports as follows: R0 (absence of cancer cells within 1 mm of all resection margins) or non-R0 (micro/macroscopic residual tumor). In cases where a patient did not undergo surgery due to distant metastases and/or locally advanced cancer on preoperative imaging on the basis of a multidisciplinary conference, the tumor was regarded to be clinically confirmed as unresectable. If histopathologic analyses were not available, the comparison between previous and follow-up images and tumor marker (CA 19–9) levels obtained for at least one year served as the reference standard.

### Statistical analysis

All statistical analyses were performed using SAS statistical software (SAS system for Windows, v9.4; SAS institute, Cary, NC), jackknife free-response receiver operating characteristic (JAFROC) software (v4.2.1), and IBM SPSS Statistics for Windows (v25.0; IBM Corp., Armonk, NY, USA). JAFROC method and generalized estimating equations were used to compare the pooled areas under the curve (AUC), sensitivity and specificity of each imaging set regarding resectability, followed by post-hoc pairwise comparison and Bonferroni correction. Cases with resectability scores 4 and 5 were regarded as resectable tumors. Interobserver agreement was evaluated using intraclass correlation coefficient (ICC) (Supplementary material 5). Kaplan–Meier method was used to estimate recurrence-free survival (RFS) in patients achieving R0 resection. Prognostic factors for RFS were assessed using univariate and multivariate Cox regression analyses. All variables with *p*-values < 0.05 in univariate analyses were included in the multivariate analysis using stepwise selection. Statistical significance was set at *p* < 0.05.

## Results

### Patient characteristics

Of the 120 patients (mean age ± standard deviation [SD], 66.7 ± 8.4 years), 65 were women (Table 1). Baseline CT resectability category determined at the multidisciplinary team (MDT) conference was resectable (14.2% [17/120]), borderline resectable (59.2% [71/120]), and locally advanced (26.7% [32/120]). Among 120 patients, 98 (81.7% [98/120]) underwent surgery (curative-intent surgery [*n* = 93], palliative due to local unresectability [*n* = 3], and palliative due to peritoneal seeding [*n* = 2]), whereas the remaining 22 (18.3% [22/120]) did not undergo surgery due to local unresectability determined at MDT conferences (*n* = 17), percutaneous biopsy-confirmed hepatic (*n* = 2) and pulmonary metastasis (*n* = 1), clinically confirmed peritoneal seeding (*n* = 1), and both local unresectability and clinically confirmed peritoneal seeding (*n* = 1). Of the 93 patients who underwent curative-intent surgery, 69 (74.2% [69/93]) achieved R0 resection.

**Table 1:** Baseline characteristics of the study population

Characteristics	Values
Age (mean ± SD, y)	66.7 ± 8.4
Sex	
Male	55 (45.8%)
Female	65 (54.2%)
Tumor location	
Head or uncinate process	73 (60.8%)
Body	37 (30.8%)
Tail	10 (8.3%)
Tumor size (mean ± SD, cm)	
Baseline	3.0 ± 1.1 (1.3–8.5)
Post-NAT	2.4 ± 1.0 (0.8–6.9)
Baseline CT resectability category	
Resectable	17 (14.2%)
Borderline resectable	71 (59.2%)
Locally advanced	32 (26.7%)
Tumor response evaluation according to RECIST 1.1	
Partial response	47 (39.2%)
Stable disease	58 (48.3%)
Progressive disease	15 (12.5%)
Serum CA 19–9 level (mean ± SD, U/mL)	
Baseline	1718.8 ± 3228.8 (1.0–12,000.0)
Post-NAT	461.3 ± 1636.5 (1.0–12,000.0)
Serum CA 19–9 response	
Responder (normalized at follow-up)	31 (25.8%)
Non-responder (nonnormalized or nonelevated CA 19–9 level)	89 (74.2%)
Types of surgery	
Pancreaticoduodenectomy	61 (50. 8%)
Distal pancreatectomy	24 (20.0%)
Total pancreatectomy	6 (5.0%)
Diagnostic laparotomy or palliative surgery	5 (4.2%)
Subtotal pancreatectomy	2 (1.7%)
No surgical procedure	22 (18.3%)
Resection margin^a^	
Negative	69 (74.2%)
Positive	24 (25.8%)
Pathologic T staging^a^	
T0 (no residual tumor)	7 (7.5%)
T1	33 (35.5%)
T2	32 (34.4%)
T3	16 (17.2%)
T4	5 (5.4%)
Pathologic N staging^a^	
N0	55 (59.1%)
N1	34 (36.6%)
N2	4 (4.3%)
Tumor grade^a^	
Well-differentiated	10 (10.8%)
Moderately differentiated	68 (73.1%)
Poorly differentiated or undifferentiated	8 (8.6%)
No residual tumor	7 (7.5%)
Tumor regression grade according to the College of American Pathologists^a^	
0 (complete response, no viable cancer cells)	7 (7.5%)
1 (near complete response, single cells or rare groups of cancer cells)	23 (24.7%)
2 (partial response, residual cancer with evident tumor regression, but more than single cells or rare groups of cancer cells)	38 (40.9%)
3 (poor or no response, extensive residual cancer with no evident tumor regression)	25 (26.9%)
Large vessel invasion confirmed on pathologic analysis^a^	7 (7.5%)
Superior mesenteric artery	2 (2.2%)
Main portal vein	2 (2.2%)
Superior mesenteric vein	1 (1.1%)
Common hepatic artery	1 (1.1%)
Celiac axis	1 (1.1%)
No large vessel invasion	86 (92.5%)
Lymphatic invasion^a^	
Yes	17 (18.3%)
No	76 (81.7%)
Microscopic vascular invasion^a^	
Yes	23 (24.7%)
No	70 (75.3%)
Perineural invasion^a^	
Yes	65 (69.9%)
No	28 (30.1%)
SUVmax at tumor-vessel contact on post-NAT PET/MRI (mean ± SD)^b^	5.0 ± 3.2 (1.5–16.3)
ADC value at tumor-vessel contact on post-NAT PET/MRI(mean ± SD, × 10^–3^ mm^2^/s)^c^	1.49 ± 0.37 (1.03–2.40)

*SD* Standard deviation, *NAT* Neoadjuvant therapy, *RECIST* Response Evaluation Criteria in Solid Tumor, *CA 19–9* Carbohydrate antigen 19–9, *PET* Positron emission tomography, *FDG* Fluorodeoxyglucose, *SUVmax* The maximum standardized uptake value, *ADC* Apparent diffusion coefficient

^a^Data are available in 93 patients who underwent curative-intent surgery

^b^Data are available in 68 patients who had mild or moderate to intense FDG uptake

^c^Data are available in 110 patients who had tumor-vessel contact; other 10 patients had resectable tumor without tumor-vessel contact

### Resectability status categorization in consensus on post-NAT CT according to NCCN guidelines and R0 resection rate of each category

According to the consensus review, 26 patients had locally advanced tumors, 34 borderline resectable tumors, 54 resectable tumors, and 6 metastatic diseases on post-NAT CT, according to the NCCN guidelines. The R0 resection rate of each category was 18.8% (6/26), 64.7% (22/34), 75.9% (41/54), and 0%, respectively.

### Changes in resectability confidence score after review of PET/MRI and CA 19–9

**Fig. 2:**
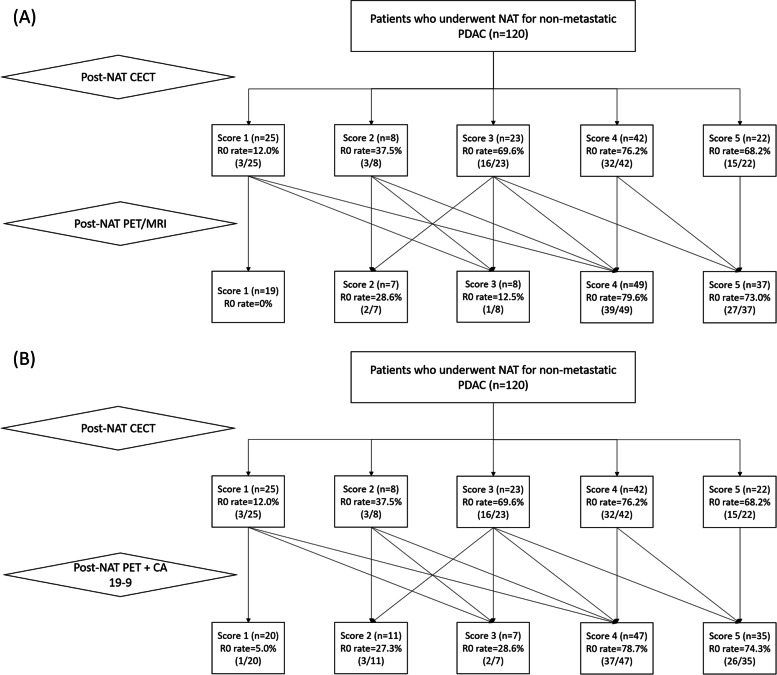
Flow diagram showing changes in CT resectability scores. Flow diagram shows changes in CT resectability scores of the three reviewers in consensus after review of post-neoadjuvant therapy PET/MRI (**A**) and PET imaging plus carbohydrate antigen 19–9 (**B**) and R0 resection rate of each score. Score 1, definitely unresectable; score 2, probably unresectable; score 3, indeterminate; score 4, probably resectable; score 5, definitely resectable. NAT, neoadjuvant therapy; PDAC, pancreatic ductal adenocarcinoma; PET, positron emission tomography; CA 19–9, carbohydrate antigen 19–9

Resectability scores in consensus among the reviewers on post-NAT CECT were distributed as: score 1 (20.8% [25/120]), 2 (6.7%, [8/120]), 3 (19.2% [23/120]), 4 (35.0%, [42/120]), and 5 (18.3% [22/120]) (Fig. 2A), and the R0 resectability of each score was 12.0% (3/25), 37.5% (3/8), 69.6% (16/23), 76.2% (32/42), and 68.2% (15/22), respectively. After addition of PET/MRI, resectability confidence scores were 1 (15.8% [19/120]), 2 (5.8% [7/120]), 3 (6.7% [8/120]), 4 (40.8% [49/120]), and 5 (30.8% [37/120]) (Fig. 2A), and the R0 resectability of each score was 0% (0/19), 28.6% (2/7), 12.5% (1/8), 79.6% (39/49), and 73.0% (27/37), respectively. Additional review of PET imaging and CA 19–9 changed the resectability confidence scores of CECT to 1 (16.7% [20/120]), 2 (9.2% [11/120]), 3 (5.8% [7/120]), 4 (39.2% [47/120]), and 5 (29.2% [35/120]) (Fig. 2B), with R0 resectability of each score being 5.0% (1/20), 27.3% (3/11), 28.6% (2/7), 78.7% (37/47), and 74.3% (26/35), respectively.

Additional review of PET/MRI led to reclassification of 24 cases with CECT scores 1, 2, or 3 towards resectability (score 4 or 5). Reclassification was most frequently observed in cases with CECT score 3 to CECT plus PET/MRI score 4 (75.0%, [18/24]) (Table 2). After addition of PET plus CA 19–9, 20 cases with CECT score 1, 2, or 3 were reclassified towards resectability, and changes in CECT score 3 to CECT plus PET plus CA 19–9 score 4 were most frequent (85.0%, [17/20]).

**Table 2:** Results of reclassification of CECT score after additional review of PET/MRI and CA 19–9

	Reclassification towards > 3		Reclassification towards ≤ 3	
CECT score	CECT plus PET/MRI	No. of cases	CECT plus PET/MRI	No. of cases
1 (*n* = 25)	4	1 (4.0%)	1	18 (72.0%)
	5	0	2	2 (8.0%)
			3	4 (16.0%)
2 (*n* = 8)	4	2 (25.0%)	1	1 (12.5%)
	5	2 (25.0%)	2	3 (37.5%)
			3	0
3 (*n* = 23)	4	18 (78.3%)	1	0
	5	1 (4.3%)	2	1 (4.3%)
			3	3 (13.0%)
4 (*n* = 42)	4	28 (66.7%)	1	0
	5	12 (28.6%)	2	1 (2.4%)
			3	1 (2.4%)
5 (*n* = 22)	4	0 (%)	1	0
	5	22 (100.0%)	2	0
			3	0
CECT score	CECT plus PET plus CA19-9	No. of cases	CECT plus PET plus CA19-9	No. of cases
1 (*n* = 25)	4	0	1	19 (76.0%)
	5	0	2	5 (20.0%)
			3	1 (4.0%)
2 (*n* = 8)	4	1 (12.5%)	1	1 (12.5%)
	5	1 (12.5%)	2	4 (50.0%)
			3	1 (12.5%)
3 (*n* = 23)	4	17 (73.9%)	1	0
	5	1 (4.3%)	2	1 (4.3%)
			3	4 (17.4%)
4 (*n* = 42)	4	29 (69.0%)	1	0
	5	11 (26.2%)	2	1 (2.4%)
			3	1 (2.4%)
5 (*n* = 22)	4	0	1	0
	5	22 (100.0%)	2	0
			3	0

*CECT* Contrast-enhanced CT, *PET* Positron emission tomography, *CA 19–9* Carbohydrate antigen 19–9

### Comparison of diagnostic performance among CECT, CECT plus PET/MRI, and CECT plus PET plus CA19-9 in determining R0 resectability

Significant differences were observed in pooled AUC of CECT, CECT plus PET/MRI, and CECT plus PET plus CA 19–9 regarding R0 resectability (0.853 vs. 0.873 vs 0.874, *p* = 0.026) (Table 3). Pairwise comparison showed that CECT plus PET plus CA 19–9 showed significantly higher pooled AUC compared to CECT (adjusted *p* = 0.047). In addition, there were significant differences in pooled sensitivity of three image sets (66.2% vs. 86.0% vs. 84.5%, *p* < 0.001). Pairwise comparison showed that sensitivities of CECT plus PET/MRI and CECT plus PET plus CA 19–9 were significantly higher than that of CECT alone (adjusted *p* < 0.001).

**Table 3:** Comparison of diagnostic performance among CECT, CECT plus PET/MRI, and CECT plus PET plus CA 19–9 and post-hoc pairwise comparison

	CECT (A)	CECT plus PET/MRI (B)	CECT plus PET plus CA 19–9 (C)	*p*-value	*p*-value*		
					A vs. B	B vs. C	C vs. A
Pooled sensitivity (%)	66.2 (137/207)	86.0 (178/207)	84.5 (175/207)	**< 0.001**	**< 0.001**	0.201	**< 0.001**
Pooled specificity (%)	67.3 (103/153)	58.8 (90/153)	60.1 (92/153)	**0.048**	**0.042**	0.945	0.081
Pooled PPV (%)	73.3 (137/187)	73.9 (178/241)	74.2 (175/236)	0.822			
Pooled NPV (%)	59.5 (103/173)	75.6 (90/119)	74.2 (92/124)	**0.001**	**< 0.001**	0.567	**< 0.001**
Pooled AUC	0.853 (0.795–0.911)	0.873 (0.802–0.944)	0.874 (0.810–0.939)	**0.026**	0.066	1.000	**0.047**

*CECT* Contrast-enhanced CT, *PET* Positron emission tomography, *CA 19–9* Carbohydrate antigen 19–9, *PPV* Positive predictive value, *NPV* Negative predictive value, *AUC* Area under the receiver operating characteristic curve

^*^Adjusted *p*-values using Bonferroni correction

**Fig. 3:**
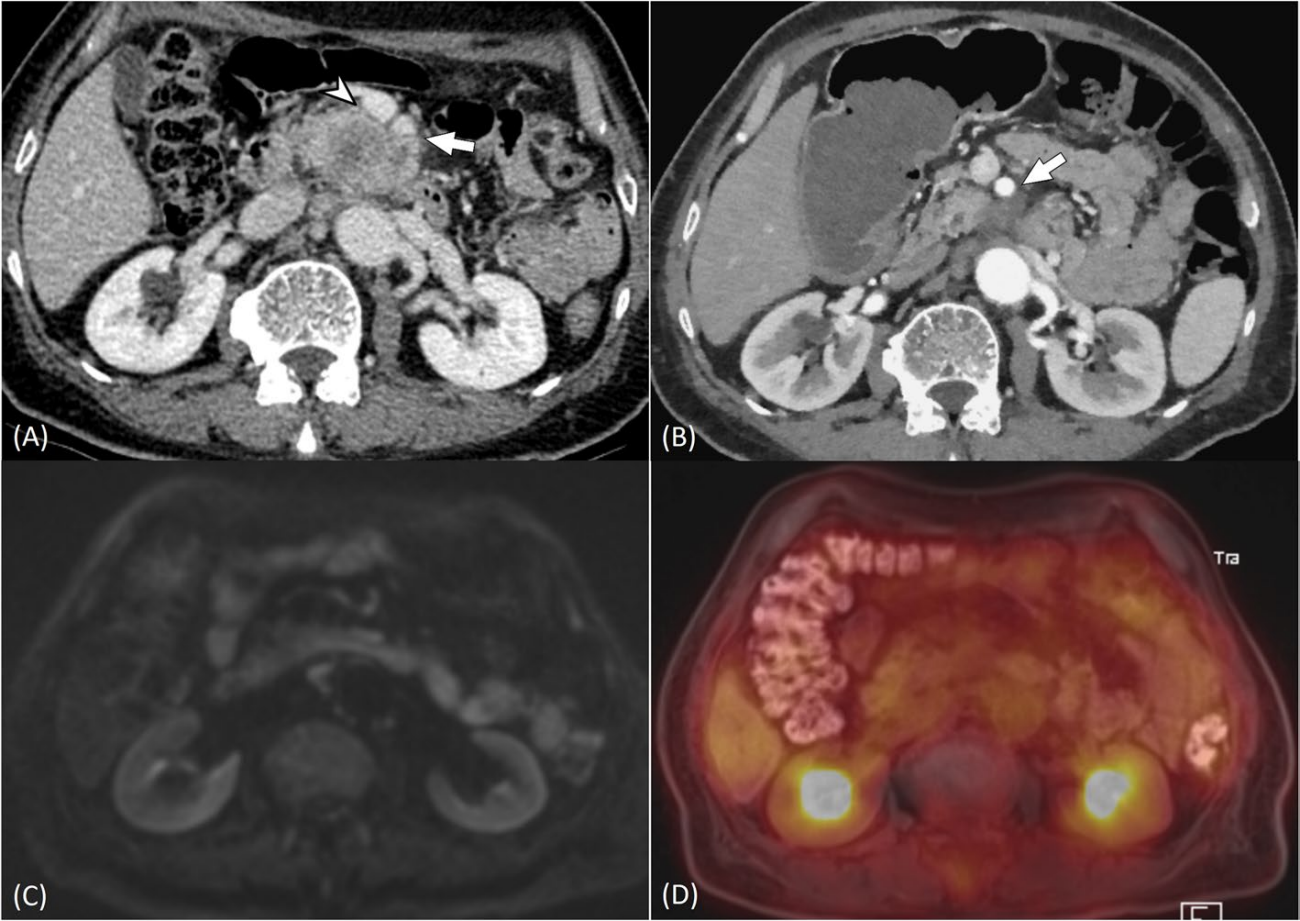
A 70-year-old man with pancreatic cancer. Axial portal venous phase image of baseline contrast-enhanced computed tomography (CECT) scan (**A**) shows a 5 cm-sized pancreatic cancer in the uncinate process, contacting > 180 degree with superior mesenteric artery (arrow) and causing contour irregularity of superior mesenteric vein (arrowhead). On arterial phase image of post-neoadjuvant therapy (NAT) CECT scan (**B**), pancreatic cancer showed decrease in size to 3 cm and ≤ 180 degree contact with superior mesenteric artery (arrow), and contour irregularity of superior mesenteric vein was resolved. CECT resectability score was 3 (indeterminate resectability) according to the three reviewers in consensus. Since there was no diffusion restriction on diffusion-weighted imaging (DWI) (**C**) and no fluorodexyglucose (FDG) avidity on. ^18^F-FDG-positron emission tomography (PET) (**D**) at tumor-vessel contact of post-NAT PET/MRI, and carbohydrate antigen level (CA) 19–9 was 3284 U/mL at initial diagnosis which reduced to 4 U/mL after NAT, the reviewers modified the resectability score to 4 (probably resectable) on both CT plus PET/MRI set and CT plus PET plus CA 19–9. The patient underwent Whipple’s surgery, and pathologic analysis showed no residual tumor with ypT0N0

**Fig. 4:**
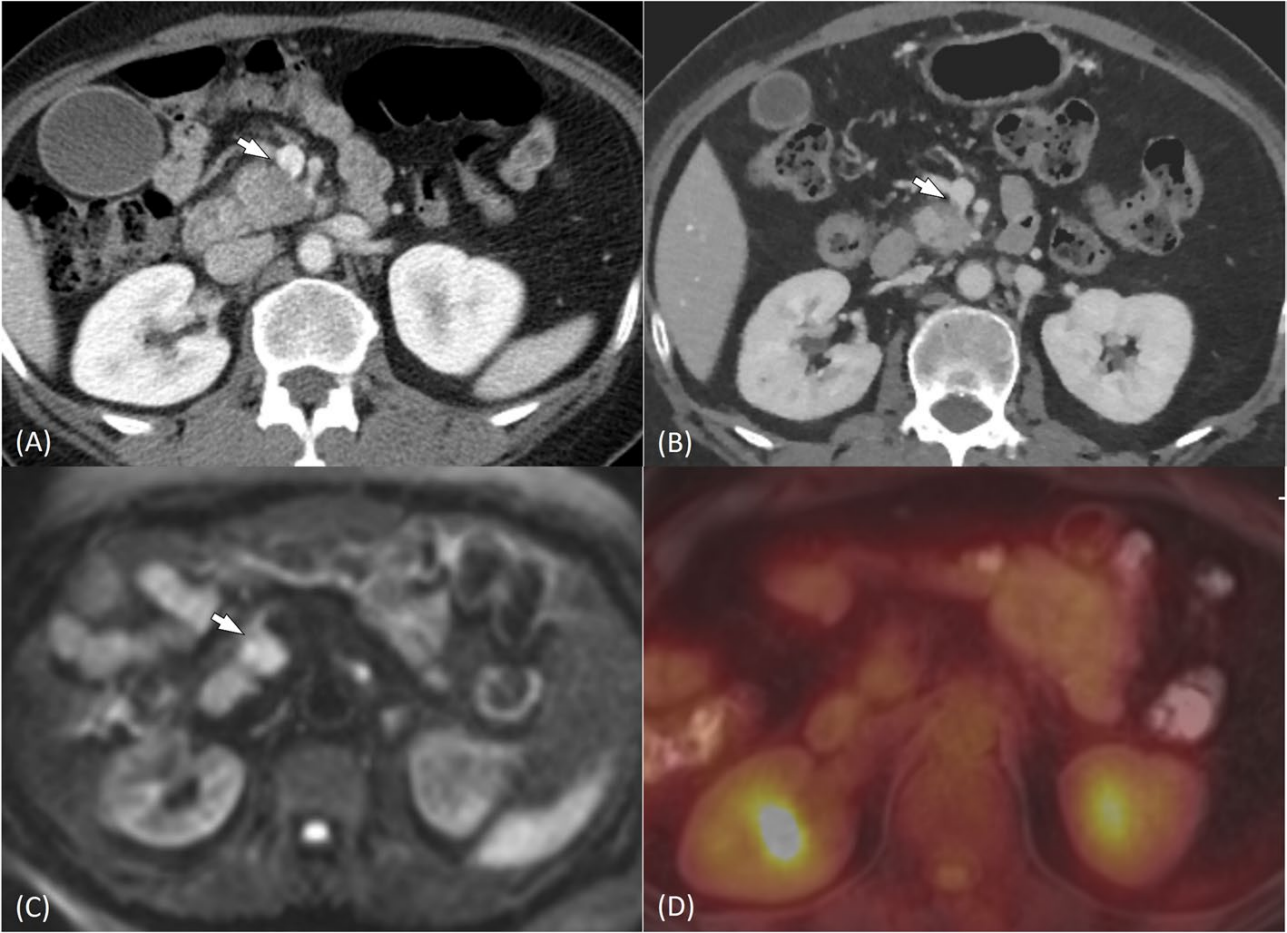
A 54-year-old woman with pancreatic cancer. Axial portal venous phase image of the baseline contrast-enhanced computed tomography (CECT) scan (**A**) demonstrates a 3 cm-sized pancreatic head cancer, contacting the superior mesenteric vein with vein contour irregularity (arrow). On arterial phase image of post-neoadjuvant therapy (NAT) CT scan (**B**), the size of pancreatic cancer decreased to 1.7 cm, but contour irregularity of the superior mesenteric vein was still noted (arrow). CECT resectability score was 3 (indeterminate resectability) according to the three reviewers in consensus. Diffusion restriction was seen at the tumor-vessel contact on diffusion-weighted imaging (DWI) (arrow) (**C**) and fluorodexyglucose (FDG) avidity at tumor-vessel contact was not seen on. ^18^F-fluorodexyglucose (FDG)-positron emission tomography (PET) (**D**) of PET/MRI. Carbohydrate antigen level (CA) 19–9 was 245U/mL at initial diagnosis, which reduced to 30 U/mL after NAT. The reviewers assigned resectability score 3 on CT plus PET/MRI set and score 4 on CT plus PET plus CA 19–9. The patient underwent Whipple’s surgery, and pathologic analysis showed College of American Pathologists grade 2 (moderate response) with ypT0N0

Significant differences were found in pooled specificity of three image sets (67.3% vs. 58.8% vs. 60.1%, *p* = 0.048) (Table 3). Specificity of CECT plus PET/MRI was marginally lower than that of CECT alone (adjusted *p* = 0.042), but there was no significant difference in specificity between CECT alone and CECT plus PET plus CA 19–9 (adjusted *p* = 0.081). Representative cases are shown in Figs. 3 and 4.

### Comparison of diagnostic performance of CECT and CECT plus PET/MRI in detection of distant metastasis

Seven patients had distant metastasis (peritoneum [*n* = 4], liver [*n* = 2], and lung [*n* = 1]). No significant differences were found between pooled AUC, sensitivity, and specificity of CECT and those of CECT plus PET/MRI to detect distant metastasis (pooled AUC, 0.877 vs. 0.983, *p* = 0.314; pooled sensitivity, 38.1% [8/21] vs. 52.4% [11/21], *p* = 0.375; pooled specificity, 97.9% [332/339] vs. 99.4% [333/339], *p* = 0.125).

### Predictive factors for RFS in patients who achieved R0 resection

Among 69 patients who achieved R0 resection, 28 (40.6% [28/69]) experienced tumor recurrence during the mean follow-up period of 18.0 ± 11.7 months (range, 0–52), which was clinically confirmed at MDT conferences (*n* = 22) or pathologically diagnosed through biopsy (*n* = 6). Tumor recurrence sites were liver (*n* = 13), local recurrence (*n* = 8), peritoneum (*n* = 5), lymph node (*n* = 3), both liver and peritoneum (*n* = 2), pleura (*n* = 1), abdominal wall (*n* = 1), both liver and lung (*n* = 1), and liver, lymph node, and peritoneum (*n* = 1). The estimated one-, three-, and five-year RFS rates were 74.6%, 44.2%, and 23.6%, respectively. FDG avidity at tumor-vessel contact on post-NAT PET (hazard ration [HR] = 2.99, 95% confidence interval [CI] = 1.36–6.55, *p* = 0.011) was significantly related to RFS, along with sex (HR = 0.46, 95% CI = 0.21–1.00), pathologic T stage (HR = 1.53, 95% CI = 1.06–2.22, *p* = 0.024), pathologic N stage (HR = 2.16, 95% CI = 1.08–4.32, *p* = 0.034), lymphatic invasion (HR = 4.08, 95% CI = 1.64–10.17, *p* = 0.006), vascular invasion (HR = 2.97, 95% CI = 1.38–6.39, *p* = 0.008), perineural invasion (HR = 2.72, 95% CI = 1.15–6.43, *p* = 0.015), tumor grade (HR = 1.59, 95% CI = 1.00–2.52, *p* = 0.048), and tumor regression grade (HR = 1.57, 95% CI = 1.01–2.43, *p* = 0.037) in univariate analyses (Table 4). Multivariate analyses showed that FDG avidity at tumor-vessel contact on post-NAT PET of PET/MRI (HR = 4.37, 95% CI = 1.13–16.92, *p* = 0.033) and vascular invasion on pathology (HR = 5.36, 95% CI = 1.73–16.59, *p* = 0.004) were independent predictors of RFS.

**Table 4:** Predictive factors for recurrence-free survival in patients who underwent R0 resection

Characteristic	Univariate			Multivariate		
	Hazard ratio	95% CI	*p*-value	Hazard ratio	95% CI	*p*-value
Gender (male)	0.46	0.21–1.00	**0.049**	0.55	0.23–1.31	0.178
Age (per 1 year)	1.02	0.97–1.07	0.472			
Baseline CT resectability category according to multidisciplinary team conference	0.49	0.24–1.00	0.051			
Post-NAT CT resectability category according to three reviewers in consensus	0.63	0.26–1.52	0.289			
Increased uptake at tumor-vessel contact on post-NAT PET of PET/MRI	2.99	1.36–6.55	**0.011**	4.37	1.13–16.92	**0.033**
ADC value < 1.40 × 10^–3^ mm^2^/s at tumor-vessel contact	1.20	0.56–2.58	0.632			
T stage^a^	1.53	1.06–2.22	**0.024**	1.48	0.87–2.52	0.152
N stage^a^	2.16	1.08–4.32	**0.034**	1.04	0.42–2.55	0.936
Lymphatic invasion^a^	4.08	1.64–10.17	**0.006**	1.94	0.64–5.85	0.239
Vascular invasion^a^	2.97	1.38–6.39	**0.008**	5.36	1.73–16.59	**0.004**
Perineural invasion^a^	2.72	1.15–6.43	**0.015**	1.16	0.29–4.64	0.836
Tumor grade^a^	1.59	1.00–2.52	**0.048**	1.28	0.72–2.30	0.402
Tumor regression grade^ab^	1.57	1.01–2.43	**0.037**	0.49	0.21–1.17	0.107
CA 19–9 response	2.00	0.75–5.34	0.139			

*CI* Confidence interval, *NAT* Neoadjuvant therapy, *PET* Positron emission tomography, *ADC* Apparent diffusion coefficient, *CA 19–9* Carbohydrate antigen 19–9

^a^Pathologically confirmed

^b^The College of American Pathologists grading systems

### Interobserver agreement of CECT, CECT plus PET/MRI, and CECT plus PET plus CA 19–9 for resectability

Interobserver agreement of CECT, CECT plus PET/MRI, and CECT plus PET plus CA 19–9 for resectability was moderate with ICC of 0.700 (95% CI, 0.621–0.770), 0.667 (95% CI, 0.582–0.743), and 0.703 (95% CI, 0.624–0.772), respectively.

Interobserver agreement for FDG avidity and diffusion restriction at tumor-vessel contact was moderate with ICC of 0.698 (95% CI, 0.622–0.771) and 0.572 (95% CI, 0.473–0.663), respectively.

## Discussion

In this study, combined review of PET imaging, CA 19–9, and CECT significantly increased sensitivity and AUC in determining resectability, compared to CECT alone, without compromising the specificity in patients who underwent NAT for PDAC. Furthermore, FDG uptake at tumor-vessel contact on PET imaging was an independent predictor for RFS after complete resection. Our study results were similar to a recent study, which demonstrated that favorable responses in both CA 19–9 and FDG-PET were necessary to achieve prognostic benefit from NAT [24], but while the previous study results focused on disease-free and overall survival, we investigated the additional information from PET imaging and CA19-9 to CECT as indicators of resectability. Since no preoperative CT nor clinical factors can accurately determine resectability after NAT, borderline resectable PDAC patients with no progression on NAT should be suggested to undergo surgical exploration [25]. Likewise, NCCN version 2022 recommended consideration of resection in patients with locally advanced PDAC, who exhibited decreased CA 19–9 level and clinical improvement [1]. However, our study revealed a R0 resection rate of only 64.7% for borderline resectable tumors, which is inferior to the R0 resection rate of 73% documented in a previous study [26]. Therefore, any further increment of confidence and accuracy regarding R0 resection on preoperative imaging compared to CECT is beneficiary.

CECT showed a tendency to overestimate residual tumor after NAT, mainly because NAT induces fibrosis rather than volume loss and leads to difficulties in characterization of residual soft tissue at the tumor-vessel contact on CECT [27]. Our study demonstrated that addition of metabolic status assessment using PET and CA 19–9 to morphologic status assessment using CECT could elevate the confidence level of physicians in determining resectability by helping differentiate residual tumor from treatment-related change, thus decreasing the number of indeterminate cases on CT, and improving prediction of R0 resection compared to CECT alone. We believe this multiparametric approach is valuable for selecting ideal surgical candidates, especially in patients having tumors with indeterminate resectability on CECT.

In our study, while CECT plus PET/MRI set showed decreased specificity, specificity of CECT plus PET plus CA 19–9 was not significantly different from that of CECT alone. The role of CA 19–9 in determining resectability in PDAC patients after NAT has been described in previous studies [20, 28], which supports our study findings. The results of these previous studies, including ours, used various cutoff values of absolute CA 19–9 level or reduction, which further demands validation of optimal cutoff. However, to the best of our knowledge, there is paucity of data on correlation between DWI and PDAC resectability [19]. In a prospective study, post-NAT whole tumor apparent diffusion coefficient value predicted R0 resectability [19]. However, our study results showed that additional review of PET and DWI decreased the specificity of CECT, which might be explained by the fact that diffusion restriction at tumor-vessel contact theoretically may not differentiate viable tumor from NAT-induced fibrosis [29].

NAT for borderline resectable and locally advanced PDAC has been associated with overall survival benefit, which could be due to downstaging of disease and greater likelihood of achieving complete resection [30]. However, tumor recurrence or metastasis occurs in majority of the cases within 1–2 years of surgery, and the median overall survival is only 20–25 months after surgery [31]. Our study results showed that FDG avidity at tumor-vessel contact on post-NAT PET/MRI and vascular invasion on pathology were independent predictors of RFS. Several studies have reported quantitative parameters of PET imaging, such as maximum standardized uptake value, metabolic tumor volume, or total lesion glycolysis to be independent prognostic factors [32–36], but those studies, in contrast to our study, included heterogeneous groups of PDAC patients (with or without NAT). Additionally, acquisition of simultaneous PET/MRI in our study might have contributed to overcoming the limitation of low spatial resolution of metabolic imaging at the crucial interface between mass and vessel. Based on our study results, we cautiously propose adjuvant chemoradiation therapy and meticulous surveillance for recurrence after surgery in patients with FDG avidity at tumor-vessel contact, irrespective of achievement of R0 resection.

Our study had few limitations. First, this was a single-center, retrospective study. PET/MRI was performed at our institution as a problem-solving tool to determine resectability in patients showing favorable response or stability after NAT, which might have led to potential selection bias and underestimation of diagnostic performance of PET/MRI. Second, MDT-based clinical decision without pathologic confirmation was used as a part of the standard of reference. Last, we did not evaluate the dynamic diagnostic contribution of MRI for resectability, but we believe both CECT and dynamic MRI may have overlapped information regarding tumor extent.

## Conclusions

Additional review of PET imaging and CA 19–9 significantly increased the sensitivity of CECT in determining R0 resectability, compared to CECT alone, without compromising specificity in patients with non-metastatic pancreatic cancer after NAT. Furthermore, FDG avidity at tumor-vessel contact on post-NAT PET of PET/MRI was an independent predictor of RFS.

## Supplementary Information

**Additional file 1: Supplementary Material 1.** Neoadjuvant chemoradiation therapy reference. **Supplementary Material 2.** CT scanners and imaging protocol. **Supplementary Material 3.** PET/MRI protocol. **Supplementary Material 4.** Post-NAT CT criteria for R0 resection. **Supplementary Material 5.** Interobserver agreement for resectability.

## Data Availability

The datasets used and/or analysed during the current study are available from the corresponding author on reasonable request.

## Abbreviations

FDG: ^18^F-fluorodeoxyglucose
PDAC: Pancreatic ductal adenocarcinoma
NAT: Neoadjuvant treatment
CA 19–9: Carbohydrate antigen 19–9
CECT: Contrast-enhanced computed tomography
AUC: Area under the curve
RFS: Recurrence-free survival
NCCN: National Comprehensive Cancer Network
HR: Hazards’ ratio
CI: Confidence interval
