# Supplementary material for: Post-neoadjuvant treatment pancreatic cancer resectability and outcome prediction using CT, 18F-FDG PET/MRI and CA 19–9
Source: Cancer Imaging. 2023 May 22;23:49. doi: 10.1186/s40644-023-00565-8 (PMC10201764; doi:10.1186/s40644-023-00565-8)
Supplement: Supplementary file 1 — Additional file 1: Supplementary Material 1. Neoadjuvant chemoradiation therapy reference. Supplementary Material 2. CT scanners and imaging protocol. Supplementary Material 3. PET/MRI protocol. Supplementary Material 4. Post-NAT CT criteria for R0 resection. Supplementary Material 5. Interobserver agreement for resectability. [file 40644_2023_565_MOESM1_ESM.docx]

**Supplementary Material 1. Neoadjuvant chemoradiation therapy reference**

Gemcitabine chemotherapy consisted of intravenous administration at a dose of 400 mg/m^2^ body surface area weekly for 6 weeks. FOLFIRINOX was composed of 400 mg/m^2^ of leucovorin and 85 mg/m^2^ of oxaliplatin both administered as a 2-hour intravenous infusion with the addition of 180 mg/m^2^ of irinotecan administered over 90 minutes as an intravenous infusion 30 minutes later. Thereafter, 500 mg/m^2^ of 5-FU was administered as an intravenous bolus followed by a continuous 2,400 mg/m^2^ infusion for 46 hours (one cycle) every 2 weeks.

Eighty-four patients underwent additional radiotherapy with 45–56 Gy in 28 fractions or stereotactic body radiotherapy with 50 Gy in five fractions.

**Supplementary Material 2. CT scanners and imaging protocol**

| CT scanner | No. of patients (%) |
| --- | --- |
| Somatom Force 192-row (Siemens Healthineers) | 29 (24.2%) |
| IQon Spectral 128-row (Philips Healthcare) | 21 (17.5%) |
| Somatom Definition 64-row (Siemens Healthineers) | 21 (17.5%) |
| Brilliance iCT 256-row (Philips Healthcare) | 17 (14.2%) |
| Ingenuity 128-row (Philips Healthcare) | 12 (10.0%) |
| Aquilion One 320-row (Canon Medical Systems) | 12 (10.0%) |
| Discovery 750 64-row (GE Healthcare) | 6 (5.0%) |
| Revolution 256-row (GE Healthcare) | 1 (0.8%) |
| Lightspeed 16-row (GE Healthcare) | 1 (0.8%) |

**Supplementary Material 3. PET/MRI protocol**

All patients fasted for at least 6 hours before the examination. The serum glucose levels were examined to ensure they were < 200 mg/dL, and each patient received an intravenous injection of FDG (5.2 MBq/kg) 60 minutes before the scan. Whole-body PET/MRI was performed from the brain to the proximal thigh. Simultaneous PET was acquired with two-point volumetric interpolated breath-hold examination (VIBE) Dixon, heavily T2-weighted imaging (T2WI), and diffusion-weighted imaging (DWI) using two b-values (0, 800sec/mm2). The axial range of each bed position was 25.8 cm, with 6.1-cm overlap between adjacent bed positions. Automatic attenuation correction of PET data was performed according to the attenuation maps generated by the Dixon VIBE sequence.

Simultaneous regional PET was acquired while performing a dedicated MRI. The total acquisition time for PET/MRI was approximately 60–80 minutes.

**Supplementary Material 4. Post-NAT CT criteria for R0 resection.**

Score 5, definitely resectable (no tumor-vessel contact); 4, probably resectable (≤180 degree contact with superior mesenteric vein [SMV] or portal vein [PV] without vein contour irregularity); 3, indeterminate probability for resectability (≤180 degree contact with celiac axis [CA], common hepatic artery [CHA], or superior mesenteric artery [SMA] or tumor contact with SMV or PV >180 degree or with contour irregularity of the vein or thrombosis of the vein but with vein reconstructibility); 2, probably unresectable (tumor contact >180 degree with the SMA, CA, or CHA or unreconstructible SMV/PV due to tumor involvement or occlusion); and 1, definitely unresectable, (tumor contact >180 degree with the SMA, CA, or CHA and unreconstructible SMV/PV due to tumor involvement or occlusion).

**Supplementary Material 5. Interobserver agreement for resectability**

ICC estimates and their 95% confidence intervals (CIs) were calculated based on a mean rating, absolute agreement, two-way mixed-effects model. Based on the 95% CI of the ICC estimate, values <0.5, between 0.5 and 0.75, between 0.75 and 0.9, and >0.90 were considered to be indicative of poor, moderate, good, and excellent reliability, respectively.
